# Assessment of the Cholesterol-Lowering Effect of MOMAST^®^: Biochemical and Cellular Studies

**DOI:** 10.3390/nu14030493

**Published:** 2022-01-23

**Authors:** Martina Bartolomei, Carlotta Bollati, Jianqiang Li, Anna Arnoldi, Carmen Lammi

**Affiliations:** Department of Pharmaceutical Sciences, University of Milan, Via Mangiagalli 25, 20133 Milan, Italy; martina.bartolomei@unimi.it (M.B.); carlotta.bollati@unimi.it (C.B.); jianqiang.li@unimi.it (J.L.); anna.arnoldi@unimi.it (A.A.)

**Keywords:** *Cultivar* Coratina, by-products, circular economy, cholesterol metabolism, PCSK9

## Abstract

MOMAST^®^ is a patented phenolic complex derived from the olive oil vegetation water, a by-product of the olive oil supply chain, in which hydroxytyrosol (OH-Tyr) and tyrosol (Tyr) and verbascoside are the main compounds. This study was aimed at investigating its hypocholesterolemic effect by assessing the ability to modulate the low-density lipoprotein (LDL) receptor (LDLR)/sterol regulatory element-binding protein 2 (SREBP-2), and proprotein convertase subtilisin/kexin type 9 (PCSK9) pathways. MOMAST^®^ inhibits the in vitro activity of 3-hydroxy-3-methylglutaryl coenzyme A reductase (HMGC_O_AR) with a dose-response trend. After the treatment of HepG2 cells, MOMAST^®^ increases the SREBP-2, LDLR, and HMGCoAR protein levels leading, from a functional point of view to an improved ability of hepatic cells to up-take LDL from the extracellular environment with a final cholesterol-lowering effect. Furthermore, MOMAST^®^ decreased the PCSK9 protein levels and its secretion in the extracellular environment, presumably via the reduction of the hepatic nuclear factor 1-α (HNF1-α). The experiments were performed in parallel, using pravastatin as a reference compound. Results demonstrated that MOMAST^®^ may be exploited as a new ingredient for the development of functional foods and/or nutraceuticals for cardiovascular disease prevention.

## 1. Introduction

The production of extra virgin olive oil (EVOO) has a significant economic value for the Mediterranean countries. In particular, Spain, Italy, and Greece are responsible for 63% of international olive annual production [1]. However, the production process has a noteworthy environmental impact, since it involves the consumption of large amounts of resources and generates emissions and water and soil pollution. More in detail, olive oil production generates huge amounts of waste, i.e., wood, branches, leaves, and by-products, such as olive pomace, olive mill wastewater, olive stones, with a relevant impact at environmental level. These residues are not only undesirable in terms of sustainability and environmental impact, but also create high management and disposal costs [2]. Nevertheless, the olive oil production by-products are rich in high-value molecules such as phenolic compounds, particularly abundant in olive oil vegetation water (OVW) [1,3]. Nowadays, there is an increasing interest in natural polyphenols given their well-known health-promoting effects, i.e., antioxidant, anti-inflammatory, anti-diabetic, anti-mutagenic, neuro-protective, and cardio-protective [4,5]. Moreover, in the area of cardiovascular diseases prevention, it has been widely demonstrated that the consumption of an EVOO rich in phenolic compounds reduces low-density lipoprotein (LDL) cholesterol (LDL-C) and triglyceride, decreases oxidative stress and lipid oxidation, and enhances the high-density lipoprotein (HDL) cholesterol (HDL-C) [6,7]. In this context, recent evidence contributes to defining the molecular mechanism at the basis of the hypocholesterolemic effect exerted by the EVOO phenolic compounds [8]. Notably, a phenolic phytocomplex extracted from the EVOO of the *Cultivar* Coratina (OMN) increases the low-density lipoprotein (LDL) receptor (LDLR) protein levels as a result of the activation of the sterol regulatory element-binding proteins (SREBP-2) transcription factor, allowing human hepatic HepG2 cells to uptake LDL particles from the extracellular environment with a final cholesterol-lowering effect [9,10].

MOMAST^®^ is a patented natural phenolic complex, rich in tyrosol (Tyr) and hydroxytyrosol (OH-Tyr), which is obtained from the OVW by-products of Coratina *cultivar* using exclusively physical and mechanical methods, without the use of solvents and other chemical processes. This sustainable product, currently on the market, is directly used in its original form or has the potential to become the main ingredient in the formulations of nutraceutical, pharmaceutical, and cosmetic products. Indeed, MOMAST^®^ has shown antioxidant and anti-inflammatory properties in rat isolated tissues after LPS stimuli [11].

In light of this evidence, the present study was aimed at fostering the pleotropic health-promoting activity of this sustainable product investigating the hypocholesterolemic properties of MOMAST^®^ through the use of in vitro and cellular techniques. In particular, dedicated experiments were performed in order to verify its direct ability to modulate in vitro the 3-hydroxy-3-methylglutaryl coenzyme A reductase (HMGCoAR) activity. In parallel, human hepatic HepG2 cells were treated with MOMAST^®^ (50 µg/mL) and western blotting experiments were carried out for assessing its effect on the modulation of the intracellular cholesterol metabolism. All the experiments were performed using pravastatin as reference compound.

## 2. Materials and Methods

### 2.1. Chemicals

More details regarding the chemicals and reagents are provided in the Supplementary Materials. Table S1 provides more detailed information regarding the products and lot number of antibodies used.

#### MOMAST^®^ Description

Bioenutra S.R.L. (Italy) supplies the patented MOMAST^®^ sample as liquid 50 mL units (production lot: B20-01/PL, 100 mg/mL) directly from the production process. Table 1 shows the main characteristics of this MOMAST^®^ sample, whereas the technical data sheets, provided by the company, are available as Supplementary Materials.

### 2.2. Cell Culture Conditions

HepG2 cell line was bought from ATCC (HB-8065, ATCC from LGC Standards, Milan, Italy) and was cultured following details which are provided in the Supplementary Materials.

### 2.3. MTT Assay

A total of 3 × 10^4^ HepG2 cells/well were seeded in 96-well plates and treated with 10.0–500 μg/mL of MOMAST^®^, or vehicle (H_2_O) in complete growth media for 48 h at 37 °C under 5% CO_2_ atmosphere. More details are provided in Supplementary Materials.

### 2.4. HMGCoAR Activity Assay

The assay buffer, NADPH, substrate solution, and HMGCoAR were provided in the HMGCoAR Assay Kit (Sigma, St. Louis, MI, USA). The experiments were carried out following the manufacturer’s instructions at 37 °C and MOMAST^®^ tested in the range of concentrations 10.0–500.0 μg/mL. More details are provided in Supplementary Materials.

### 2.5. Western Blot Analysis

Experiments were performed using protocol and conditions previously optimized [10]. Further details are reported in Supplementary Materials.

### 2.6. In-Cell Western (ICW) Assay

A total of 3 × 10^4^ HepG2 cells/well were seeded in 96-well plate and, the following day, they were treated with 50.0 μg/mL MOMAST^®^ or Pravastatin 1.0 µM in complete growth medium for 24 h. See Supplementary Materials for further details.

### 2.7. Fluorescent Cell-Based LDL Uptake

The experiments were carried out following the manufacturer’s instructions. See Supplementary Materials for further details.

### 2.8. Quantification of Secreted Proprotein Convertase Subtilisin/Kexin Type 9 (PCSK9)

The supernatants, collected from HepG2 cells previously treated with 50.0 μg/mL of MOMAST^®^, were centrifuged at 600× *g* for 10 min at 4 °C. They were recovered and diluted with the ratio of 1:10 with DMEM in a new ice-cold tube. The experiments were carried out at 37 °C following the manufacturer’s instructions. The absorbance at 450 nm was measured using Synergy H1 fluorescent plate reader (Biotek, Winusky, VT, USA). More details are reported in Supplementary Materials.

### 2.9. Statistically Analysis

All the data set were statistically analyzed following the procedure reported in Supplementary Materials.

## 3. Results

### 3.1. Effect of MOMAST^®^ on the HMGC_O_AR Activity

**Figure 1:**
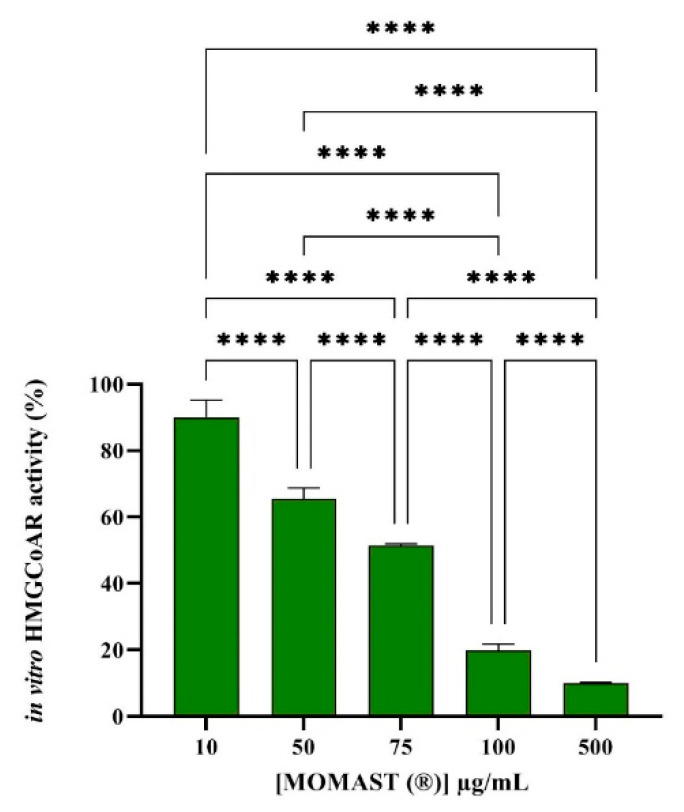
Effect of MOMAST^®^ on the HMGCoAR activity. The data points represent the averages ± SD of three experiments in triplicate, (****) *p* < 0.0001 vs. control (C). HMGCoAR: 3-hydroxy-3-methylglutaryl coenzyme A reductase.

The HMGCoAR, a key enzyme in the intracellular cholesterol biosynthesis, is the target of statins, the main drugs employed for the hypercholesterolemia management and control [12,13]. Based on this consideration, for assessing the MOMAST^®^ capacity to modulate the HMGCoAR activity, a biochemical investigation was carried out. Findings suggest that MOMAST^®^ inhibits the enzyme activity with dose response trend (Figure 1), since it drops the enzyme activity by 10 ± 5.2%, 34.5 ± 3.2%, 48.5 ± 0.5%, 80.2 ± 1.9%, and 90 ± 0.3%, at 10.0, 50.0, 75.0, 100.0, and 500.0 µg/mL, respectively.

### 3.2. Effect of MOMAST^®^ on the HepG2 Cell Vitality

**Figure 2:**
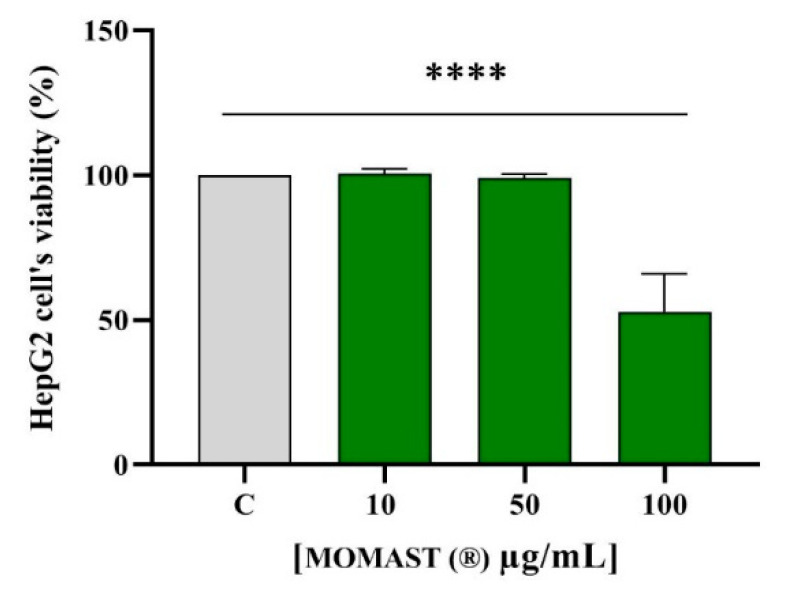
Effect of MOMAST^®^ on HepG2’s cell viability. The data points represent the averages ± SD of three experiments in triplicate, (****) *p* < 0.0001 vs. control (C).

MTT experiments were conducted for sorting out those concentrations of MOMAST^®^ that may potentially determine cytotoxic effects on HepG2 cells. Notably, no cytotoxic effect was observed up to 50.0 µg/mL, after 48 h treatment, whereas at 100.0 µg/mL a 47.2 ± 3.4% cell mortality was observed (Figure 2). Hence, the following experiments performed at the cellular level were carried out at concentrations equal to 50.0 µg/mL.

### 3.3. Effect of MOMAST^®^ on the LDLR Pathway Modulation

**Figure 3:**
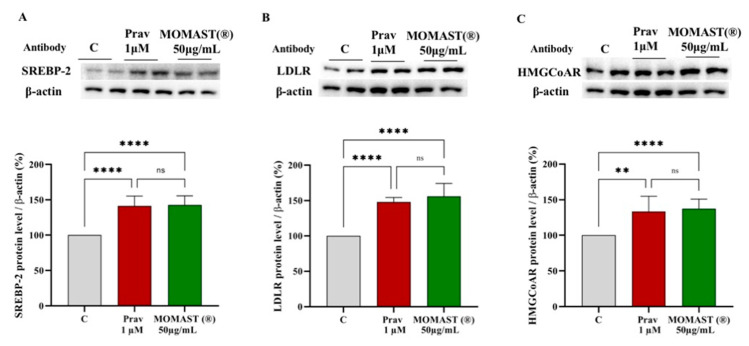
Effect of MOMAST^®^ (50.0 μg/mL) and Pravastatin (Prav) 1.0 μM on SREBP2 (Panel (**A**)), LDLR (Panel (**B**)), and the HMGCoAR (Panel (**C**)) protein levels. Bars represent the averages of three independent experiments performed in duplicate ± SD, ns: not significant, (**) *p* < 0.01, (****) *p* < 0.0001 vs. control (C). SREBP-2: sterol regulatory element-binding proteins; LDLR: low-density lipoprotein; HMGCoAR: 3-hydroxy-3-methylglutaryl coenzyme A reductase.

To evaluate the modulating effects on cholesterol metabolism, western blotting experiments were carried out on cell lysates of HepG2 cells treated with MOMAST^®^ (50.0 μg/mL) and with pravastatin (1 μM) as a reference control. Figure 3 indicates that MOMAST^®^ increased the level of the SREBP-2 transcription factor protein by 142.8% ± 12.8% (*p* < 0.0001, Figure 3A) and this increase led to an improvement of total LDLR and HMGCoAR proteins up to 155.9 ± 18.2% (*p* < 0.0001, Figure 3B) and 137.4 ± 13.5% (*p* < 0.0001, Figure 3C), respectively.

In the same set of experiments, pravastatin (1 µM), improved the SREBP-2 protein level by 156 ± 4.6% (*p* < 0.0001, Figure 3A), the LDLR by 152 ± 19.7% (*p* < 0.0001, Figure 3B), and the HMGCoAR protein by 133.4 ± 12.4% (*p* < 0.01, Figure 3C). From a statistical point of view, no significant difference was observed between MOMAST^®^ and pravastatin.

### 3.4. Functional Effect of MOMAST^®^ on the Ability of HepG2 Cells to Uptake LDL from the Extracellular Environment

**Figure 4:**
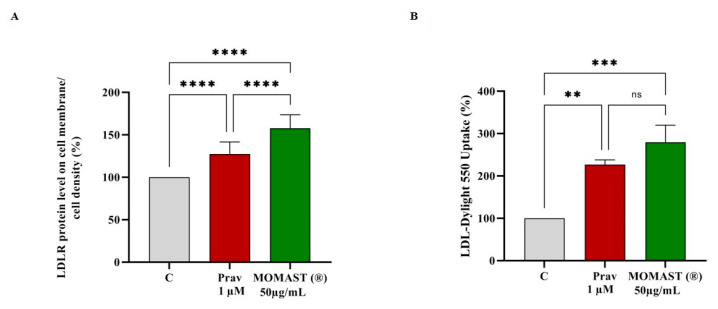
HepG2 cells were treated with MOMAST^®^ (50.0 μg/mL) and Pravastatin (Prav) 1.0 μM for 24 h. The percentage of LDLR protein level was measured by ICW (Panel (**A**)). The data points represent the averages ± SD of three experiments in duplicate. (****) *p* < 0.0001 vs. control (C). (Panel (**B**)) Effect of MOMAST^®^ on the HepG2 cell ability to uptake LDL. The data points represent the averages ± SD of three experiments in triplicate. ns: not significant, (**) *p* < 0.01, and (***) *p* < 0.001 vs. control (C). LDLR: low-density lipoprotein receptor, LDL: low-density lipoprotein.

The ability of MOMAST^®^ to modulate the protein level of LDLR on the HepG2 cell surface was assessed by using an ICW assay, i.e., a quantitative colorimetric cell-based assay that allows the detection of target proteins in fixed cultured cells [14]. An improvement of the LDLR levels specifically localized on the cellular membrane of hepatocytes, up to 157.5 ± 16.2% (*p* < 0.0001) was observed (Figure 4A). In the same set of experiments, at 1 µM, the positive control pravastatin increased the LDLR by 127.3 ± 14.3% (*p* < 0.0001. Figure 4A). The increase of membrane LDLR protein levels led to an improved functional ability of HepG2 cells to absorb the extracellular LDL by 279.6 ± 40.1% after the treatment with MOMAST^®^ at the same concentration of 50 µg/mL (*p* < 0.001, Figure 4B). In the same conditions, pravastatin (1 µM) improves the ability of hepatic cells to uptake LDL by 226.9 ± 11.1% from the extracellular space (*p* < 0.01, Figure 4B).

### 3.5. Effect of MOMAST^®^ on the Modulation of PCSK9 Pathway

**Figure 5:**
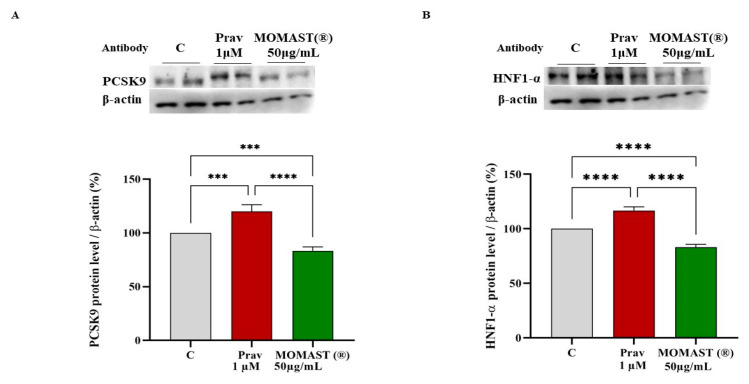
Impact of MOMAST^®^ on PCSK9 (Panel (**A**)) and the HNF1-α (Panel (**B**)) protein levels. Bars represent the averages of three independent experiments performed in duplicate ± SD (***) *p* < 0.001 and (****) *p* < 0.0001 vs. control (C). Prav: pravastatin. PCSK9: proprotein convertase subtilisin/kexin type 9; HNF1-α: hepatic nuclear factor 1-α.

To evaluate the effect of MOMAST^®^ on the modulation of PCSK9 and its transcription factor HNF1-α, HepG2 cells were treated at 50.0 µg/mL and, in parallel, with pravastatin (1 μM) as a positive control. Figure 5 indicates that MOMAST^®^ caused a reduction of intracellular PCSK9 protein levels of 16.7 ± 3.8% towards untreated cells (*p* < 0.001 Figure 5A), this finding agrees with the ability of MOMAST^®^ to reduce the HNF1-α transcription factor levels by 16.9 ± 2.5% compared to untreated cells (*p* < 0.0001 Figure 5B). Pravastatin (1 μM), on the other hand, increases the levels of this target up to 120.1 ± 6.2% (*p* < 0.001 Figure 5A) and this agrees with the increase of the HNF1-α transcription factor up to 116.7 ± 3, 4 (*p* < 0.0001, Figure 5B).

**Figure 6:**
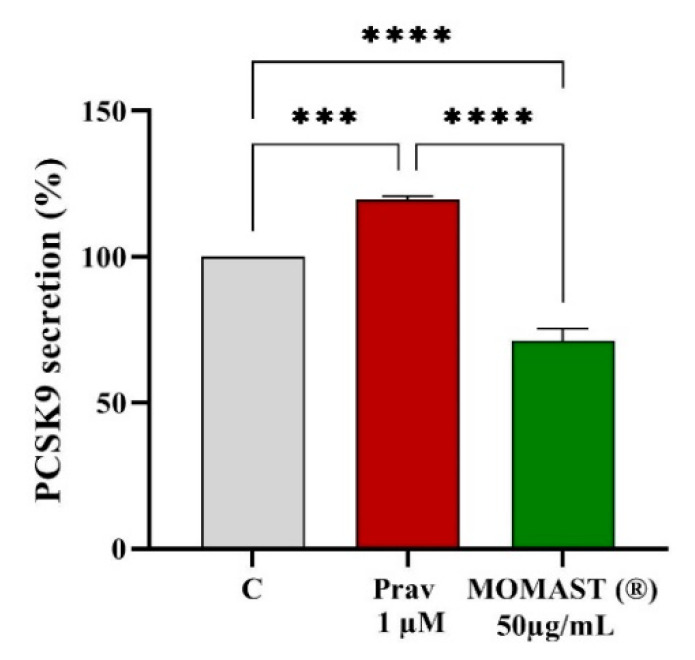
Impact of MOMAST^®^ on the PCSK9 secretion level. Data points represent averages ± SD of three independent experiments in duplicate. (***) *p* < 0.001 and (****) *p* < 0.0001 vs. control (C). Prav: pravastatin. PCSK9: proprotein convertase subtilisin/kexin type 9.

Based on the results, the effect of MOMAST^®^ on the modulation of mature PCSK9 secretion by HepG2 cells was evaluated by ELISA assay. In agreement with molecular results, MOMAST^®^ reduces the secretion of the mature PCSK9 by 28.7 ± 4.1%, (*p* < 0.0001, Figure 6), while pravastatin increases PCSK9 secretion up to 119.6 ± 1.2% (*p* < 0.001, Figure 6).

## 4. Discussion

The beneficial effects of OMW extracts have been demonstrated both in cellular and in vivo studies, in particular in the field of cancer prevention, cellular aging in neurodegenerative diseases, and in the prevention of coronary diseases [2]. Notably, clear evidence suggests that the OH-Tyr purified from OMW and OMW extract exert hypocholesterolemic effects in rats fed with a cholesterol-rich diet [15]. Indeed, the administration of a low-dose (2.5 mg/kg of body weight) of OH-tyr and a high-dose (10 mg/kg of body weight) of OMW extract significantly lowered the serum levels of total cholesterol (TC) and low-density lipoprotein cholesterol (LDL-C) while increasing the serum levels of high-density lipoprotein cholesterol (HDL-C) [15].

In this context, MOMAST^®^ is a patented phenolic extract of OMW from Coratina *cultivar* rich in OH-Tyr, Tyr, and verbascoside. Our study provides a deeper mechanistic investigation demonstrating how MOMAST^®^ modulates cholesterol metabolism. The results clearly suggest that this product significantly reduces the HMGCoAR activity in a dose-dependent manner. Hence, this effect prompted us to investigate in a detailed way the cellular modulation of the LDLR pathway, upon HMGCoAR inhibition.

The cellular study was carried out on HepG2 cells because the hepatocyte is the major cell involved in the clearance of plasma LDL cholesterol expressing the most numbers of active LDLR on its surface [16]. In addition, since HepG2 expresses differentiated hepatic functions, these cells are worldwide recognized as a good model for assessing the hypocholesterolemic effects of bioactive compounds from different sources [17,18,19,20,21].

Preliminary MTT experiments were carried out to exclude any potential cytotoxic effect of MOMAST^®^ showing that it is safe for HepG2 cells up to 50 µg/mL. Based on these results, hepatic cells were treated with 50 µg/mL of MOMAST^®^. Similarly, to pravastatin (1 µM, the positive control), by inhibiting the HMGCoAR activity, MOMAST^®^ modulated the intracellular cholesterol pathway, leading to an increase of the LDLR and HMGCoAR protein levels through the modulation of SREBP-2 (Figure 3 and Figure 4). By using ICW assay, it was demonstrated that MOMAST^®^ increases the LDLRs which are localized on the surface of HepG2 cells leading from a functional point of view to an increased ability of hepatic cells to absorb LDL from the extracellular environment with a final hypocholesterolemic effect (Figure 4).

The molecular behavior of MOMAST^®^ is similar to pravastatin (Figure 3 and Figure 4), however, a different effect was observed on the PCSK9 pathway. PCSK9 is a secreted protein, which binds the LDLR expressed on the surface of the hepatocytes [16] and the PCSK9-LDLR binding activates the receptor catabolism leading to the hepatic LDLR degradation. Indeed, a promising strategy for the development of a new hypocholesterolemic drug consists of the PCSK9 inhibition and/or modulation [22]. Interestingly since both PCSK9 and LDLR contain functional sterol regulatory elements (SREs) in their promoters, both these proteins are co-regulated by SREBP-2 [23,24]. However, since the HNF1-α binding site is unique to the PCSK9 promoter and is not present in the LDLR promoter, the modulation of the PCSK9 transcription through HNF1-α does not affect the LDLR pathway. Thus, the co-regulation of PCSK9 from LDLR and other SREBP target genes is disconnected by the HNF1-α binding site [24,25]. In this context, it is important to highlight that statins increase the PCSK9 expression, which dampens an effective LDL clearing by promoting LDLR degradation [26], thereby counteracting the therapeutic effects of these drugs [23].

In light with these considerations, our results confirm that pravastatin increases the mature PCSK9 protein levels through the augmentation of HNF1-α, on the contrary, MOMAST^®^ significantly reduces HNF1-α leading to a decrease of PCSK9 levels. In agreement with these results, it was observed that only MOMAST^®^, and not pravastatin, is able to reduce the secretion of PCSK9 in the extracellular environments. In fact, MOMAST^®^ decreased its secretion by 28.7 ± 4.1%, whereas pravastatin increased it by 19.6 ± 1.2%. This result contributes to explain the better ability of MOMAST^®^ to raise the level of LDLR population localized on the surface of HepG2 cells than pravastatin. In fact, as shown in Figure 5A, MOMAST^®^ is significantly more effective than pravastatin in the LDLR increment. Therefore, taking together all these results, it clearly appears that MOMAST^®^ is able to mediate a complementary cholesterol-lowering effect, overcoming the intrinsic limits of the mechanism of action of statin.

Recently, we have demonstrated that OMN displays a hypocholesterolemic effect with a molecular mechanism that is similar to MOMAST^®^ [10]. OMN contains OH-Tyr (16.7%) and Tyr (30.8%) and other secoiridoidic compounds, i.e., oleuropein and ligstroside and their derivatives. Through the comparison of their effects on the modulation of the key target involved in the cholesterol metabolism pathway, it appears clear that both samples modulate the LDLR pathway via the activation of SREBP-2 transcription factors, showing similar behavior, however, only MOMAST^®^ is able to modulate the PCSK9 pathway, displaying the complementary cholesterol-lowering mechanism of action. Doubtlessly, this difference may be explained considering the different compositions of these phytocomplexes, even though from a functional point of view, their biological effects are comparable. Thus, our results support the functional role of both OH-Tyr and Tyr within a phytocomplex for exerting the hypocholesterolemic effect.

## 5. Conclusions

Since the exploitation of by-products is becoming more and more important in recent years, MOMAST^®^ represents a useful and sustainable alternative to solve the OVW problems, through the valorization of OVW derived phenolic extract. Notably, this study provides clear evidence regarding the cholesterol-lowering effect of MOMAST^®^ which can be used as an ingredient for the development of new dietary supplements and or functional food for the prevention of cardiovascular disease. In light of these preliminary in vitro results and with the aim at fostering practical exploitation of this ingredient, further in vivo study will be realized.

## 6. Patents

Patent n. 102021000019226 of the 20 July 2021 entitled “Processo produttivo di complessi polifenolici da acque di vegetazione olearie con processo fermentativo e relativi complessi polifenolici prodotti”–inventors: Arnoldi A., Clodoveo M. L., Corbo F.F.R., Franchini C., Lammi C., Lentini, G. Lorenzo, V., Massari C.D, Milani G., Moretti P., Pisano I.

## Figures and Tables

**Table 1:** MOMAST^®^ sample features and phenols composition.

Name	Type	Phenols Composition (%)	Appearance
MOMAST^®^	PLUS 30	OH-Tyr = 4.35	Red liquid
		Tyr = 0.95	
		Verbascoside = 0.46	
		Total polyphenols = 6.47	

OH-Tyr: hydroxytyrosol; Tyr: tyrosol.

## Data Availability

Not applicable.

## Supplementary Materials

The following supporting information can be downloaded at: www.mdpi.com/article/10.3390/nu14030493/s1, technical and analysis data sheets of MOMAST^®^, Table S1: product and lot number of antibodies used.
