# Supplementary material for: Assessment of the Cholesterol-Lowering Effect of MOMAST®: Biochemical and Cellular Studies
Source: Nutrients. 2022 Jan 23;14(3):493. doi: 10.3390/nu14030493 (PMC8838113; doi:10.3390/nu14030493)
Supplement: Supplementary file 1 [file nutrients-14-00493-s001.zip › nutrients-1555335-supplementary.pdf]

Technical data and analysis sheets of MOMAST® provided by Bioenutra S.r.l.

BIOENUTRA S.r.l.
TECNOLOGIE INNOVATIVE PER L'UOMO E L'AMBIENTE
Sede stabilimento: IV Traversa dx S.P. Bandiera, snc – 74013 Ginosa (+39TA)
Tel. +390994502234 – email: info@bioenutra.com – www.bioenutra.com

PRODUCT SPECIFICATIONS: MOMAST Plus BIO – Liquid

(Product from Organic raw material)

GENERAL SPECIFICATIONS	
**CAS Nr. 84012-27-1** *(OLEA EUROPAEA FRUIT EXTRACT)*	**MOMAST Plus BIO: Polyphenolic complex rich in hydroxityrosol and other polyphenols (Liquid)**
**Appearance**	Brown to dark brown slightly viscous Liquid
**Total polyphenols content** *(HPLC)*	**> 60 g/kg (See Details in Biophenolic Composition Table)**
**Loss On Drying (%)**	~ 45%
**Pesticides Residue**	Negative
**Bulk Density**	~ 1,2 kg/dm^3
**Microbial Limits:**<br>*- Total Aerobic Microbial Count*<br>*-Total Combined Yeast & Molds count*<br>*- E. Coli*<br>*- Salmonella Sp.*<br>*- S. Aureus*	 **≤ 10^3 CFU/g**<br>**≤ 10^2 CFU/g**<br>**Absent / 10 g**<br>**Absent / 10 g**<br>**Absent / 10 g**

BIOPHENOLIC COMPOSITION TABLE
(Last HPLC analysis: 15/3/2021)

Main Active molecules	Specifications	Production lot: B20-01/PL
Hydroxytyrosol	4-6%	4,35%
Tyrosol	0,4-1,5%	0,95%
Verbascoside	0,02-0,5%	0,46%
Oleuropein	0,0-1%	0,02%
Other olives polyphenols	0,5-3,5%	0,68%
Total Polifenols	>6,0%	6,47%

Scheda Tecnica Prodotto MOMAST® Plus BIO
Revisione: 31.10.2021 **Versione:** 2.0
Data di stampa: 31.10.2021

Product name
MOMAST Plus BIO *- Complesso polifenolico ricco in idrossitirosolo ed altri polifenoli da olive biologiche – Liquido*

Produttore/fornitore
BIOENUTRA SRL
Paese d'origine
Italia
Regione
Puglia

Natura del Prodotto: **Naturale**
Pianta: **OLEA EUROPEA**
Parte: **Frutto**
Condizioni di crescita: Coltivata
Periodo di raccolta materia prima: Ottobre - Marzo

INCI: OLEA EUROPAEA (OLIVE) FRUIT EXTRACT
CAS NUMBER: 84012-27-1 EINECS: n/a
Codice doganale: 1302 1300 90

Tipo di prodotto: Preparato di origine vegetale per uso nutraceutico
Aroma Naturale

Composizione qualitativa
Ingredienti: Acqua, estratto di olive (frutto)

Composizione percentuale indicativa
4-6% idrossitirosolo
0,4-1,5% tirosolo
0,02-0,5% verbascoside
0,0-1% oleuropeina
0,5-3,5% altri polifenoli dell'oliva
20-30% zuccheri ed altri componenti naturalmente presenti nel frutto dell'oliva
30-50% acqua

CARATTERISTICHE CHIMICO FISICHE TIPICHE
Aspetto: liquido, base acquosa
Pinta usata: acque di vegetazione ricavate dalla lavorazione in prima spremitura delle olive della varietà Coratina (cultivar autoctona pugliese), provenienti da coltivazioni biologiche.
Colore: marrone
Odore: caratteristico intenso
Ph: 5-5.5
Intervallo punto di ebollizione: 98-102°C
Densità a 20°C: 1.2 g/cm3

Pag. 1 a 2

Scheda Tecnica Prodotto MOMAST® Plus BIO
Revisione: 31.10.2021 **Versione:** 2.0
Data di stampa: 31.10.2021

Solubilità: solubile in acqua
Altri solventi: solubile in alcool e glicerina
Carica batterica totale < 100 ufc/g
Patogeni: assenti
Conducibilità: 22680 µS/cm
Polifenoli totali: >60 g/kg

RACCOMANDAZIONI DI UTILIZZO
Il prodotto è indicato per l'utilizzo nutraceutico quale ingrediente attivo con proprietà antiossidanti o come Aroma Naturale in preparati alimentari.
Concentrazioni di utilizzo indicate: secondo le esigenze del responsabile della formulazione
Conservare in luogo fresco ed evitare l'esposizione prolungata all'aria, al calore ed alla luce. Evitare l'utilizzo in associazione a sostanze ossidanti.
Il prodotto ha Ph acido.

NOTE
Preparato di origine vegetale conforme al Decreto Ministeriale 9/7/2012 e 10/8/2018
Aroma Naturale conforme al Reg. UE 1334/2008 (AROMI ED INGREDIENTI AROMATIZZANTI)
Esente da allergeni alimentari (Reg. 1169/2011/UE)
Esente da OGM (Reg. 1829-1830/2003/CE)
Materia Prima proveniente da agricoltura biologica
Non contiene derivati di origine animale
Nel processo produttivo non si impiegano solventi organici
Indicato per vegetariani e vegani
Adatto alla dieta Halal e Kosher

CONTATTI
Bioenutra S.R.L.
IV Traversa dx S.P. Bandiera, snc
Ginosa (TA) 74013
info@bioenutra.com
+39 099 4502234

Pag. 2 a 2

Table S1. Product and lot number details of antobodies used.

AB	*Lot. N*	*Product*
BACTIN	07M4799V	A5441
HMGCoAR	GR211866-24	Ab174830
LDLR	VB2951773A	PA5-22976
PCSK9	41752	GTX129859
HNF1α	41080	GTX 113850
SREBP2	K1913	Sc-13552

Data File C:\Chem32\1\Data\2021-03-15\Momast PLUS L B20-01 2021-03-15 14-55-15.D
Sample Name: Momast PLUS L B20-01

===

Acq. Operator : SYSTEM
Sample Operator : SYSTEM
Acq. Instrument : HPLC 1220   Location : 1
Injection Date : 3/15/2021 3:11:13 PM
Inj Volume : Manually
Acq. Method : C:\CHEM32\1\METHODS\Q-SHORT-2019.M
Last changed : 3/15/2021 1:18:01 PM by SYSTEM
(modified after loading)
Analysis Method : C:\CHEM32\1\METHODS\Q-SHORT-2019.M
Last changed : 3/15/2021 3:48:29 PM by SYSTEM
(modified after loading)
Sample Info : Momast PLUS L B20-01
Campione: 0.025mg+ 0.6 mg S.I.+ 1.3Meoh

Sample-related custom fields:

Name	Value
Additional Info : Peak(s) manually integrated	

===

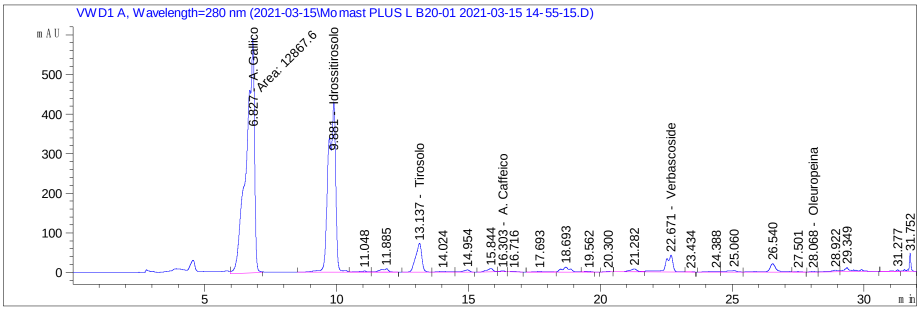

===

Internal Standard Report

===

Sorted By : Signal
Calib. Data Modified : 3/15/2021 3:47:21 PM
Multiplier : 1.0000
Dilution : 80.0000
Use Multiplier & Dilution Factor with ISTDs
Sample ISTD Information:

ISTD #	ISTD Amount [mg/kg]	Name
1	300.00000	A. Gallico

===

Acq. Operator : SYSTEM
Sample Operator : SYSTEM
Acq. Instrument : HPLC 1220 Location : 1
Injection Date : 3/15/2021 3:11:13 PM
Inj Volume : Manually
Acq. Method : C:\CHEM32\1\METHODS\Q-SHORT-2019.M
Last changed : 3/15/2021 1:18:01 PM by SYSTEM
(modified after loading)
Analysis Method : C:\CHEM32\1\METHODS\Q-SHORT-2019.M
Last changed : 3/15/2021 3:48:29 PM by SYSTEM
(modified after loading)
Sample Info : Momast PLUS L B20-01
Campione: 0.025mg+ 0.6 mg S.I.+ 1.3Meoh

Sample-related custom fields:

Name	Value
Additional Info : Peak(s) manually integrated	

===

Signal 1: VWD1 A, Wavelength=280 nm

RetTime [min]	Type	ISTD used	Area [mAU*s]	Amt/Area ratio	Amount [mg/kg]	Grp	Name
6.827	MM I	1	1.28676e4	1.00000	2.40000e4		A. Gallico
9.881	BV R	1	8114.22949	2.87595	4.35253e4		Idrossitirosolo
13.137	BB	1	1163.19373	4.38726	9518.29981		Tirosolo
16.303	VB	1	33.20021	0.00000	0.00000		A. Caffeico
22.671	VV R	1	808.54376	3.07737	4640.84889		Verbascoside
28.068	BB	1	16.12370	7.28050	218.94723		Oleuropeina

Totals without ISTD(s) : 5.79034e4

Uncalibrated Peaks : using compound Idrossitirosolo

RetTime [min]	Type	ISTD used	Area [mAU*s]	Amt/Area ratio	Amount [mg/kg]	Grp	Name
11.048	VV E		61.35201	9.07951e-2	10.38974		?
11.885	VB E		184.95044	1.96623	678.27248		?
14.024	BV		49.11551	0.00000	0.00000		?
14.954	VB		89.37666	9.70752e-1	161.82520		?
15.844	BV		143.59846	1.69815	454.82086		?
16.716	BB		22.58212	0.00000	0.00000		?
17.693	BV E		53.56312	0.00000	0.00000		?
18.693	VV R		286.33420	2.29585	1226.11487		?
19.562	VB E		25.67476	0.00000	0.00000		?
20.300	BB		16.83798	0.00000	0.00000		?
21.282	BV E		143.47691	1.69714	454.16404		?
23.434	VB E		8.00803	0.00000	0.00000		?
24.388	BV		48.25426	0.00000	0.00000		?
25.060	VB		85.04366	8.72600e-1	138.41118		?
26.540	BV R		369.71191	2.43146	1676.65890		?

===

Acq. Operator : SYSTEM
Sample Operator : SYSTEM
Acq. Instrument : HPLC 1220 Location : 1
Injection Date : 3/15/2021 3:11:13 PM
Inj Volume : Manually
Acq. Method : C:\CHEM32\1\METHODS\Q-SHORT-2019.M
Last changed : 3/15/2021 1:18:01 PM by SYSTEM
(modified after loading)
Analysis Method : C:\CHEM32\1\METHODS\Q-SHORT-2019.M
Last changed : 3/15/2021 3:48:29 PM by SYSTEM
(modified after loading)
Sample Info : Momast PLUS L B20-01
Campione: 0.025mg+ 0.6 mg S.I.+ 1.3Meoh

Sample-related custom fields:

Name	Value
Additional Info	Peak(s) manually integrated

===

RetTime [min]	Type	ISTD used	Area [mAU*s]	Amt/Area ratio	Amount [mg/kg]	Grp	Name
27.501	VB E		14.99064	0.00000	0.00000		?
28.922	BV		90.28904	9.90218e-1	166.75536		?
29.349	VB		251.28638	2.21199	1036.72870		?
31.277	BV E		37.76133	0.00000	0.00000		?
31.752	VBAR		221.97673	2.12151	878.34961		?

Uncalib. totals : 6882.49096

10 Warnings or Errors :

Warning : Negative results set to zero (cal. curve intercept)
Warning : Negative results set to zero (cal. curve intercept), (A. Caffeico)
Warning : Negative results set to zero (cal. curve intercept)
Warning : Negative results set to zero (cal. curve intercept)
Warning : Negative results set to zero (cal. curve intercept)
Warning : Negative results set to zero (cal. curve intercept)
Warning : Negative results set to zero (cal. curve intercept)
Warning : Negative results set to zero (cal. curve intercept)
Warning : Negative results set to zero (cal. curve intercept)
Warning : Negative results set to zero (cal. curve intercept)

===

*** End of Report ***
